# Aaron Douglas (1899–1979). Noah's Ark, 1927

**DOI:** 10.3201/eid0901.000000

**Published:** 2003-01

**Authors:** Marcia Smalls, Polyxeni Potter

**Affiliations:** * Centers for Disease Control and Prevention, Atlanta, Georgia, USA

**Figure:**
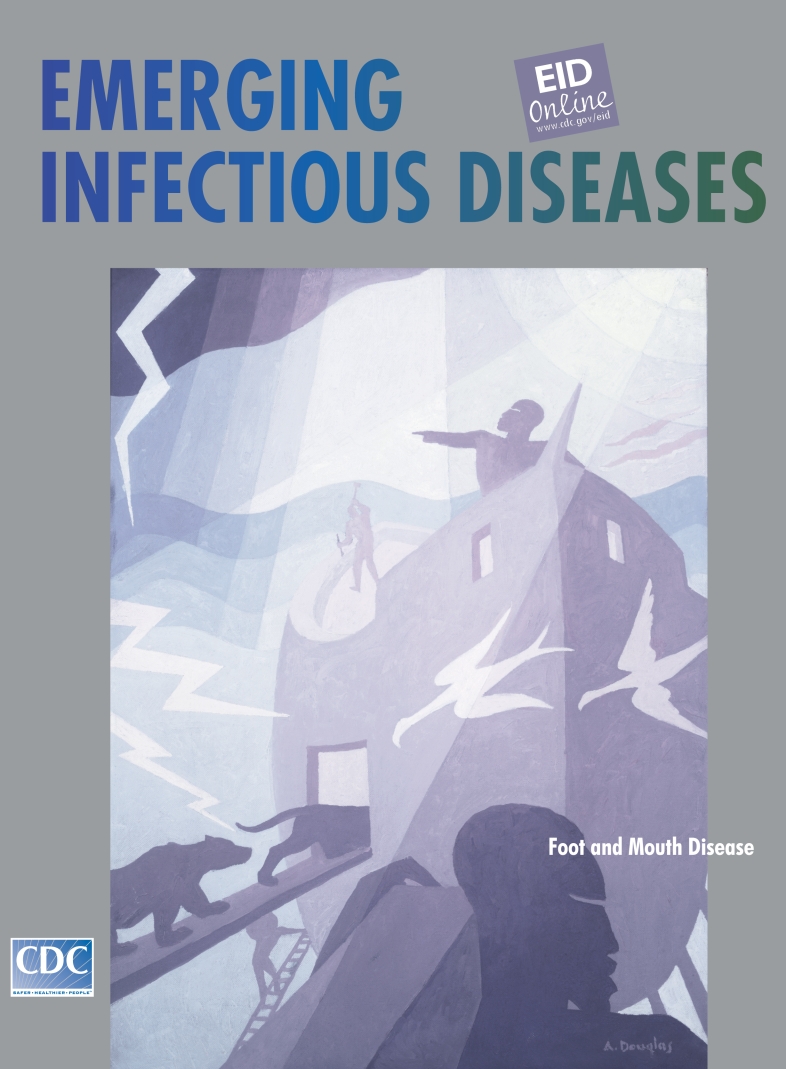
**Aaron Douglas (1899–1979). Noah’s Ark, 1927.** Oil on masonite, 4’ X 3’ Fisk University Galleries, Nashville, Tennessee

Aaron Douglas, a native of Kansas, studied art in Nebraska before going to Paris and finally settling in New York City, where he became part of the flourishing art scene of the 1920s and 1930s known as Harlem Renaissance. Douglas drew inspiration from this powerful cultural movement that encompassed all fields of art and advocated celebration of African cultural identity and heritage (*1*).

Douglas, whose diverse artistic inclinations included cubism (a stylistic invention of Georges Braque and Pablo Picasso) and African sculpture, was greatly influenced by German-born American artist Winold Reiss (1886–1953). Reiss, son of celebrated architectural designer Fritz Reiss, was a master painter of folk themes. His work captured ethnic and local characteristics in portraits of powerful universal appeal and showcased Pueblo and African cultures in the same way Paul Gauguin’s work showcased Far Eastern culture (*2*). Reiss encouraged Aaron Douglas to bring cultural identity to the forefront of his art.

In a series of seven paintings based on a book of poems by James Weldon Johnson, God’s Trombones: Seven Negro Sermons in Verse, Douglas successfully breathed cultural life into work inspired by cubism and African sculpture motifs. Primitive and mystic elements added intensity and complexity to these modern compositions and enhanced their multidimensional scope.

Noah’s Ark, the painting featured on this month’s cover of Emerging Infectious Diseases, is characterized by the formal, analytical innovations of cubism. A narrow range of sober hues (greens, beiges, blues, whites) allows uninterrupted concentration on the strict geometric definition of space. The transparent, overlapping geometric forms define the desired perspective. The ark is thrust to the foreground, preceded only by the prominent African mask, which firmly anchors the viewer into a geographic and chronological milieu. Sharp lightening strikes, terse animals heading for cover, and a focused crew advancing the vessel’s final course bespeak highest emergency.

Cataclysmic disasters fill the pages of human history, from Moses to “the little Dutch boy.” The stuff of nightmares, these disasters touch a chord because they reach beyond individual tragedy to massive plight of global proportions. Like other universal themes, the ark derives its appeal from broad applicability: haven of last resort protects those inside from impending disaster for the greater good.

Our times, plagued by (among other pestilences) the relentless emergence of global communicable disease, have constructed their own version of the ark, quarantine. But in this modern version, those sealed inside the vessel are not saved; they perish for the common good. Modern quarantine (as practiced in the prevention of mad cow disease, foot and mouth disease, and other epizootics) protects the whole from the damaged parts. Still, the infected are sealed in the ark to ensure long-term survival of the herd.
